# Berberine moderates glucose metabolism through the GnRH-GLP-1 and MAPK pathways in the intestine

**DOI:** 10.1186/1472-6882-14-188

**Published:** 2014-06-09

**Authors:** Qian Zhang, Xinhua Xiao, Ming Li, Wenhui Li, Miao Yu, Huabing Zhang, Fan Ping, Zhixin Wang, Jia Zheng

**Affiliations:** 1 Key Laboratory of Endocrinology, Ministry of Health, Department of Endocrinology, Peking Union Medical College Hospital, Peking Union Medical College, Chinese Academy of Medical Sciences, Beijing 100730, China

**Keywords:** Diabetes, Digestive tract, Gene expression, GnRH

## Abstract

**Background:**

Berberine is known to improve glucose and lipid metabolism disorders, but it poorly absorbed into the blood stream from the gut. Therefore, the exact underlying mechanism for berberine is still unknown. In this study, we investigated the effect of berberine on glucose metabolism in diabetic rats and tested the hypothesis that berberine acts directly in the terminal ileums.

**Methods:**

Rats were divided into a control group, diabetic group (DM), low dose of berberine group (BerL) and high dose of berberine group (BerH). Ileum samples were analyzed using a Roche NimbleGen mRNA array, qPCR and immunohistochemistry.

**Results:**

We found that 8 weeks of treatment with berberine significantly decreased fasting blood glucose levels. An oral glucose tolerance test (OGTT) showed that blood glucose was significantly reduced in the BerL and BerH groups before and at 30 min, 60 min and 120 min after oral glucose administration. Plasma postprandial glucagon-like peptide-1 (GLP-1) levels were increased in the berberine-treated groups. The ileum from the BerH group had 2112 genes with significantly changed expression (780 increased, 1332 decreased). KEGG pathway analyses indicated that all differentially expressed genes included 9 KEGG pathways. The top two pathways were the MAPK signaling pathway and the GnRH signaling pathway. Q-RT-PCR and immunohistochemistry verified that glucagon-like peptide 1 receptor (Glp1r) and mitogen activated protein kinase 10 (Mapk10) were significantly up-regulated, in contrast, gonadotropin releasing hormone receptor (Gnrhr) and gonadotropin-releasing hormone 1 (Gnrh1) were down-regulated in the BerH group.

**Conclusion:**

Our data suggest that berberine can improve blood glucose levels in diabetic rats. The mechanisms involved may be in the MAPK and GnRh-Glp-1 pathways in the ileum.

## Background

Diabetes is a disorder of the metabolism of carbohydrates, lipids and proteins in which the body cannot produce insulin or cannot use it to its full potential. Over 135 million people are affected by diabetes worldwide [1]. Diabetes can cause long-term complications such as retinopathy, neuropathy and nephropathy, and people with diabetes are at higher risk of myocardial infarction, stroke and limb amputation. Diabetes therapy is centered upon the control of blood glucose levels. Management of hyperglycemia with few side effects remains a challenge to the medical system.

*Rhuzima Coptidis* was recorded as an anti-diabetes medication approximately 1500 years ago in a book titled “Note of Elite Physicians” by Hongjing Tao. Berberine is the major active component of *Rhizoma coptidis*, and many studies have been published on the glucose reducing mechanisms of berberine. Zhou et al. found that berberine stimulated glucose transport through a mechanism distinct from insulin in 3T3-L1 adipocyes [2]. Moreover, berberine could activate AMPK and induced glycolysis in L6, C2C12, and 3 T3-L1 cell lines [3]. Additionally, berberine dose-dependently inhibited respiration in L6 myotubes through its specific effect on the respiratory complex I [4].

Berberine has beneficial properties in lipid and glucose metabolism regulation and has been effectively used in the treatment of diabetes, obesity and hypercholesterolemia. However, berberine has a low bioavailability [5], which is attributed to its poor aqueous solubility and dissolution.

Thus, we hypothesized that berberine can change gene expression in the intestine directly to moderate lipid and glucose metabolism. To understand the exact mechanisms, we used a type 2 diabetes rat model to investigate differential expression of genes in the intestine.

## Methods

### Animal modeling, groups, and treatment

Male Sprague–Dawley rats (280–320 g) were purchased from the Institute of Laboratory Animal Science, Chinese Academy of Medical Sciences and Peking Union Medical College (Beijing, China, SCXK-2012- 0007). According to a previous study [6] diabetic rats were fed a high-fat diet (40% of calories as fat) for 4 weeks and then administered with a single dose of streptozotocin (STZ, 50 mg/kg, tail vein) formulated in 0.1 mmol/l citrate buffer, pH 4.5 (Sigma-Aldrich, Germany). One week after the STZ injection, the random blood glucose levels of the diabetic rats were measured to confirm hyperglycemia. Random blood glucose measurements above 16.7 mmol/l were used to define rats as diabetic. Diabetic rats were fed a high-fat diet throughout the experiment. Diabetic rats with a similar degree of hyperglycemia were randomly divided into three groups: vehicle, low dose berberine (BerL), and high dose berberine (BerH) groups (n = 8, in each group). The typical human daily dose of berberine is 1200 mg/60 kg body weight. According to the formula: d_rat_ = d_human_ X 0.71/0.11 [7], the corresponding dose of berberine for rats is 129.09 mg/kg per day, so we selected 120 mg/kg and 240 mg/kg per day as low and high dosages, respectively. The control (n = 8) and vehicle group received 0.5% saline, whereas the BerL and BerH groups were given berberine at 120 mg/kg and 240 mg/kg in 0.5% saline, respectively. The drug was administered once daily for 8 weeks using a gastric gavage. All animals were housed in an environmentally controlled room at 25°C in a 12 h light: 12 h darkness cycle and given free access to food and water throughout the experimental period. Fasting animals were allowed free access to water. After 6 weeks of treatment, an oral glucose tolerance test (OGTT) was performed. After 8 weeks of treatment, blood samples were taken from the rats after anesthesia. The rats were then sacrificed. Some terminal ileum sample was collected to perform the microarray and quantitative real-time PCR (qRT-PCR) experiments. Some terminal ileum sample was fixed in 10% neutralized formalin for immunohistochemical staining. All procedures involving animals were approved by the animal care and use committee of the Peking Union Medical College Hospital (Beijing, China, MC-07-6004) and were conducted in compliance with the Guide of the Care and Use of Laboratory Animals (NIH publication No. 86-23, revised 1996). All surgeries were performed under sodium pentobarbital anesthesia, and all efforts were made to minimize suffering.

### Measurement of body weight and fasting blood glucose levels

Body weight was monitored every 2 weeks. The 6-h fasting blood glucose (FBG) level was measured monthly using the enzyme end-point method (Roche, Germany) with blood from a tail bleed.

### Oral glucose tolerance test (OGTT)

After the rats fasted for 6 h, 2.2 g/kg of glucose was orally administered. Then, blood samples were collected from tail veins at 0 min (prior to glucose load), 30 min, 60 min, and 120 min (after glucose load) for the glucose assay. The area under the curve (AUC) was calculated for blood glucose during the OGTT using the following equation: AUC = 0.5 × (BG0 + BG30)/2 + 0.5 × (BG30 + BG60)/2 + 1 × (BG60 + BG120)/2, where BG is blood glucose.

### Serum biochemistry analysis

At week 6, after the rats fasted for 6 h, the animals were euthanized. Rats received 2.2 g/kg of glucose by gavage, then the abdominal cavity was opened and a canula was inserted in the portal vein. Before the glucose load, some blood samples were taken and then centrifuged at 1000 g for 10 min. Serum was stored in aliquots at -80°C for an assay of serum fasting insulin. At 15 min following the glucose load, portal blood samples were collected in EDTA tubes containing dipeptidyle peptidase IV inhibitor (10 μL/mL blood sample, Millipore, MA, USA) via the portal vein. Plasma samples were obtained and stored at -80°C for assessment of glucagon-like peptide-1 (GLP-1). Serum insulin and plasma active GLP-1 (i.e., GLP-1 [7-36 amide] and GLP-1 [7-37]) was measured with an ELISA (Millipore, USA). HOMA-IR = FBG (mmol/L) × FINS (μU/mL)/22.5.

### RNA preparation and whole-genome gene expression profiling array experiments

The terminal ileum was taken from the BerH group and DM group (n = 3, in each group) to perform the microarray experiments. We selected the Rat 12 × 135 K Gene Expression Array which was manufactured by Roche NimbleGen (Germany). This array includes approximately 26,420 genes. Before the microarray experiment, total RNA was harvested using TRIzol (Invitrogen, CA, USA) and an RNeasy kit (Qiagen, Valencia, CA, USA) according to the manufacturer’s instructions. Total RNA from each sample was quantified by the NanoDrop ND-1000 and RNA integrity was assessed by standard denaturing agarose gel electrophoresis. The total RNA of each sample was labeled using a NimbleGen One-Color DNA labeling kit and hybridized in a NimbleGen Hybridization System. After hybridization and washing, the processed slides were scanned with an Axon GenePix 4000B microarray scanner (Molecular Devices Corporation, USA). The microarray experiment was independently repeated in triplicate.

### Gene array data analysis

The data files were imported into Agilent GeneSpring Software (Agilent, version 11.0, USA) for analysis. The gene expression level in the BerH group was normalized to that in the DM group. Differentially expressed genes were identified through Fold Change and *t*-test *P*-value screening.

To assign biological meaning to the group of genes with changed expression, the subset of genes that met the above criteria was analyzed with the Gene Ontology (GO) classification system, using Database for Annotation, Visualization, and integrated Discovery (DAVID) software (http://http:david.abcc.ncifcrf.gov/) [8] as well as the Kyoto Encyclopedia of Genes and Genomes (KEGG). All thresholds in our analyses were set to 0.001. Over-representation of genes with altered expression within specific GO categories was determined using the one-tailed Fisher exact probability modified by the addition of a jack-knifing procedure, which penalizes the significance of categories with very few (*eg.* one or two) genes and favors more robust categories with larger numbers of genes [9].

### Quantitative real time PCR analysis

For validation of the microarray results, quantitative real time PCR (Q-RT-PCR) analyses were performed using SYBR Green. Each Q-RT-PCR assay was repeated using three biological replicates and each analysis consisted of three technical replicates. Before PCR, each total RNA was processed with RNase-free DNase (Qiagen, Valencia, CA, USA). RNA was reverse transcribed by Superscript II (Invitrogen, CA, USA). The primers were designed using Applied Biosystems (Foster City, CA, USA) Primer Express™ design software. Primers were purchased from Applied Biosystems (Table 1). The reaction production could be accurately measured in the exponential phase of amplification by the ABI prism 7700 Sequence Detection System, with the following cycling conditions: an initial denaturation at 48°C for 30 min, 95°C for 15 min, 40 cycles of 95°C for 15 sec, 55°C for 1 min, and a final unlimited 4°C hold. The sequences of the primers used are listed in Table 1. The signal of the housekeeping gene glyceraldehyde-3-phosphate dehydrogenase (*Gapdh*) was used for normalization. Relative quantification of the mRNA between BerL, BerH and DM rats was calculated with the comparative Ct method [10].

**Table 1:** Olgonucleotide sequences for Q-PCR

**Gene symbol**	**Forward primer**	**Reverse primer**
*Glp1r*	CATCGTGGTATCCAAACTGA	GCTCGTCCATCACAAAGG
*Gnrh1*	TGGTATCCCTTTGGCTTTCA	TCCTCCTCCTTGCCCATCTC
*Gnrhr*	TTGTTGATGGCTGAGCAGTGA	AAGCCCGTCCTTGGAGGAAAT
*Mapk10*	TCGAGACCGTTTCAGTCCAT	CCACGGACCAAATATCCACT
*Gadph*	GACCCCTTCATTGACCTCAAC	CGCTCCTGGAAGATGGTGATG

*Glp1r*: glucagon-like peptide 1 receptor; *Gnrh1*: gonadotropin releasing hormone 1; *Gnrhr*: gonadotropin releasing hormone receptor; *Mapk10*: mitogen activated protein kinase 10.

### Immunohistochemical straining

Ileum samples from the BerH and DM groups (n = 6 in each group) were fixed in 10% neutral buffered formalin, cast in paraffin, sliced into 4-μm sections and placed onto microscope slides. After the removal of the paraffin by xylene and dehydration by graded alcohol, the slides were immersed into distilled water. Ileum sections were then transferred into a 10 mmol/L citrate buffer solution (pH 6.0) and heated at 80°C for 5 min for antigen retrieval. After washing, 3.0% peroxide was applied for 20 min to block the activity of endogenous peroxidase. To avoid nonspecific staining, the sections were incubated in blocking solution (5% BSA, Sigma, Germany) for 1 h at room temperature, followed by treatment with rabbit polyclonal anti-GLP1R antibody (1:100, Abcam lnc., UK), or rabbit polyclonal anti-MAPK10 antibody (1:100, Abcam Inc., UK), where indicated, overnight at 4°C. Negative control sections were stained under identical conditions by substituting the primary antibody with equivalent concentrations of normal rabbit IgG. After washing with phosphate-buffered saline, the slides were incubated with a labeled streptavidin biotin reagent, following the manufacturer’s instructions. Immunoreactive products were visualized with the DAB reaction. Sections were counterstained with hematoxylin for 15 sec. Brownish yellow granular or linear deposits were interpreted as positive areas. Three observers who were blinded to the clinical information evaluated the immunohistochemical staining scores independently. Staining intensity was graded semi-quantitatively using the H-SCORE [11], which was calculated using the following equation: H-SCORE = ∑Pi (i + 1), where i is the intensity of staining with a value of 1, 2 or 3 (mild, moderate, or strong, respectively) and Pi is the percentage of epithelial cells stained with different intensity, varying from 0% to 100%. The results are expressed as the mean ± SE. Differences between the groups were statistically analyzed with a one-way analysis of variance (ANOVA). A *P* value of < 0.05 was considered significant.

### Statistical analysis

All results are expressed as the mean ± standard deviation (SD). Statistical analyses were performed with analysis of variance followed by Student’s *t-*test. *P* < 0.05 was considered statistically significant. Analysis was done with SPSS 11.0 (SPSS, Inc., Chicago, IL, USA).

## Results

### Berberine showed no effect on body weight of DM rats

**Figure 1:**
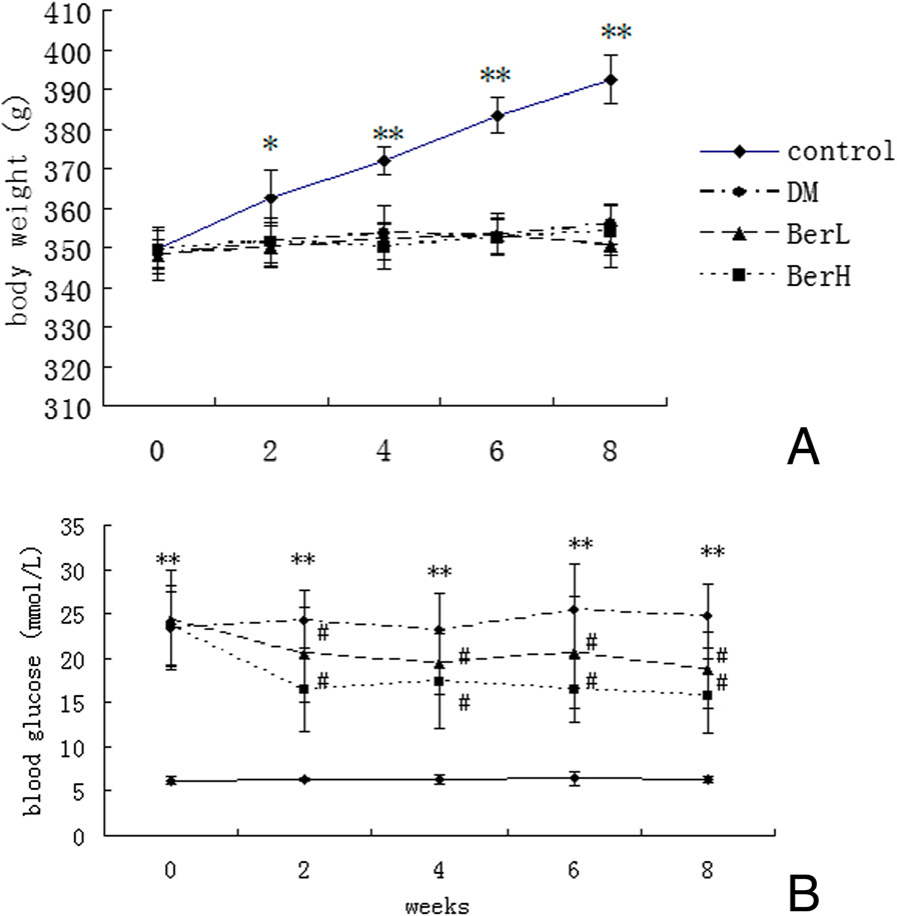
**The body weight (A) and fasting blood glucose (B) before and after berberine treatment in the rats (n = 8, in each group).** Data represent mean ± S. D. (n = 8). *P < 0.05, **P < 0.01 versus the control group; #*P* < 0.05 versus DM group.

The mean body weight of diabetic rats was significantly decreased compared to the control rats at week 2 (*P* < 0.05), week 4 (*P* < 0.01), week 6 (*P* < 0.01) and week 8 (*P* < 0.01). No significant differences were noted between the DM group and berberine-treated groups (Figure 1A).

### Berberine decreased fasting blood glucose of DM rats

The fasting blood glucose (FBG) levels of DM rats and the BerL group and BerH group were significantly higher than those of control rats at week 0 (*P* < 0.01), week 2 (*P* < 0.01), week 4 (*P* < 0.01), week 6 (*P* < 0.01) and week 8 (*P* < 0.01). FBG in the BerL group and BerH group decreased significantly at week 2 (*P* < 0.05), week 4 (*P* < 0.05), week 6 (*P* < 0.05) and week 8 (*P* < 0.05) compared to the DM group (Figure 1B).

### Berberine moderated the glucose tolerance of DM rats

**Figure 2:**
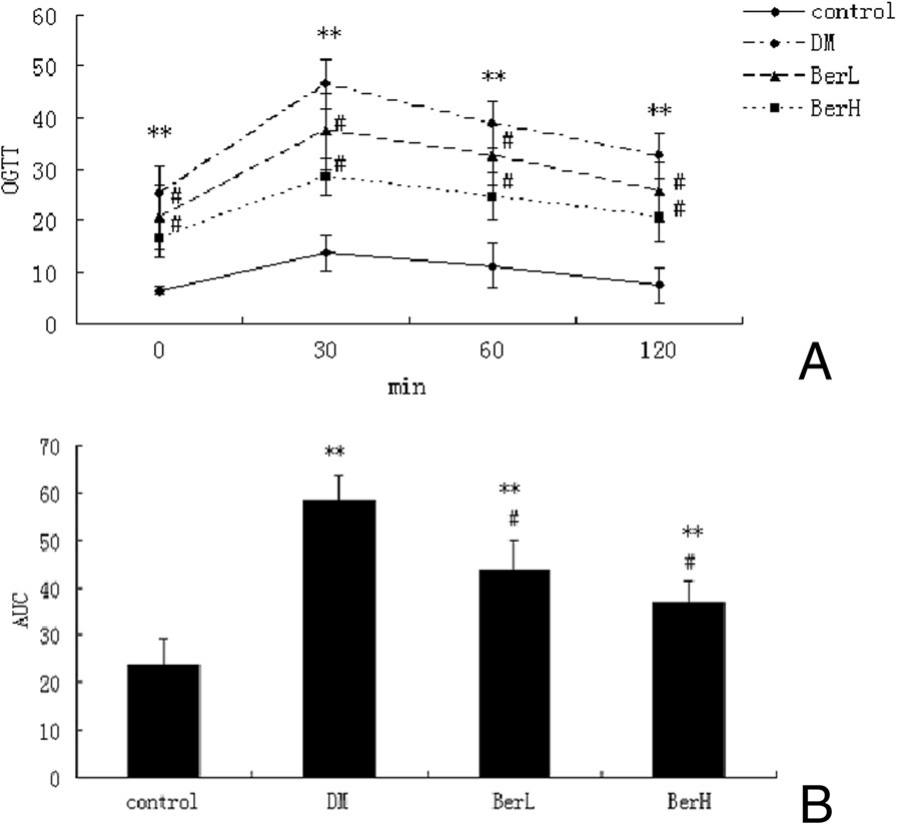
**The effect of berberine on oral glucose tolerance test blood glucose (A) and AUC (B) in rats (n = 8, in each group).** Data represent mean ± S. D. (n = 8). **P < 0.01 versus the control group; #*P* < 0.05 versus DM group.

The blood glucose levels of the DM, BerL and BerH groups were higher than those of the control group before (0 min, *P* < 0.01) and at 30 min (*P* < 0.01), 60 min (*P* < 0.01) and 120 min (*P* < 0.01) after oral glucose administration. Blood glucose levels of the BerL and BerH groups significantly decreased before (0 min, *P* < 0.05) and after oral glucose administration (*P* < 0.05 all above, Figure 2A). The AUC of OGTT for the DM, BerL and BerH groups increased compared to the control group (*P* < 0.05). The AUC for the BerL and BerH groups reduced compared with the DM group (*P* < 0.05, Figure 2B).

### Berberine reduced FINS and HOMA-IR in DM rats

**Figure 3:**
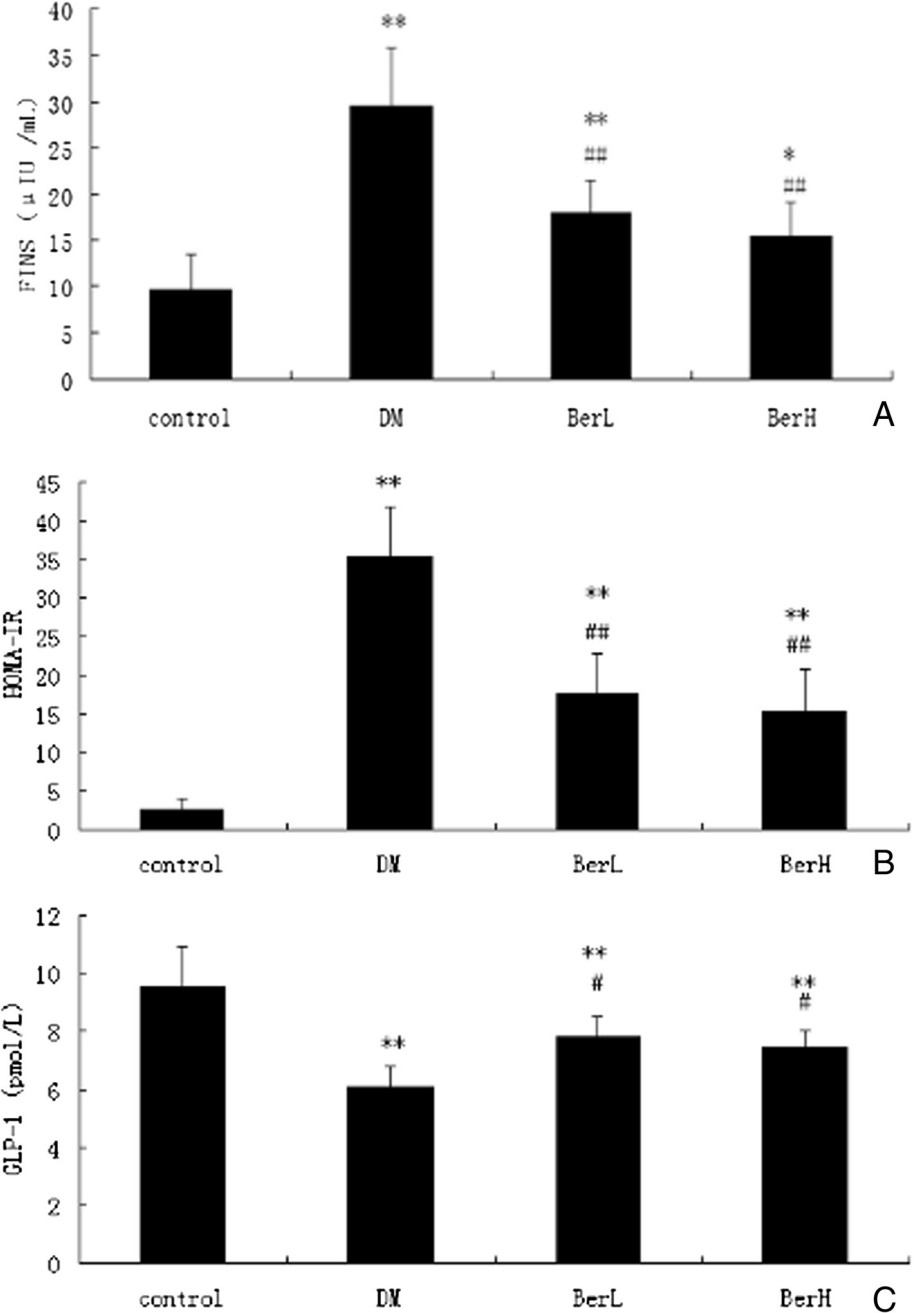
**The effect of berberine on serum fasting insulin (A), HOMA-IR (B) and plasma postprandial GLP-1 (C) in rats (n = 8, in each group).** Data represent mean ± S. D. (n = 8). **P < 0.01 versus the control group; # *P* < 0.05, ##*P* < 0.01 versus DM group.

FINS and HOMA-IR levels of the DM rats were significantly elevated (*P* < 0.01) after 8 weeks of treatment and berberine significantly suppressed FINS (*P* < 0.05) and HOMA-IR (*P* < 0.01, Figure 3A and B).

### Berberine increased GLP-1 in DM rats

Plasma postprandial GLP-1 levels in the BerH group were increased to the DM group (Figure 3C).

### Genes differentially regulated by berberine

There were 2,112 differentially expressed genes identified in the terminal ileum between the BerH group and DM group. Of those, 780 genes (36.9%) were up-regulated and 1,332 genes (63.1%) were down-regulated in the BerH group.

The aforementioned DAVID annotation tool was used for identification of GO and putative KEGG pathways. DAVID analysis of all the differentially expressed genes yielded 55 GO categorues (FDR<0.001, Table 2). The genes were mapped to 9 pathways (FDR < 0.001, Table 3). Two of the most common types of enriched pathways were the MAPK signaling pathway (FDR = 3.26E-19) and the GnRH signaling pathway (FDR = 1.01E-13). In the MAPK signaling pathway, in particular, 266 genes were represented on the gene array, of which 95 genes were differentially expressed.

**Table 2:** Gene ontology groups with significant over-representation among genes with significantly changed expression in the BerH (FDR < 0.001)

**GO classification**	**Go term**	**GO ID**	**Count**	**Fold enrichment**	**FDR**
Biological process	Intracellular signaling cascade	GO:0007242	60	7.195	1.61E-18
	Phosphate metabolic process	GO:0006796	55	7.186	1.06E-17
	Phosphorus metabolic process	GO:0006793	55	7.169	1.20E-17
	Protein kinase cascade	GO:0007243	34	14.126	7.10E-16
	Regulation of phosphorylation	GO:0042325	37	10.287	1.03E-15
	Regulation of phosphate metabolic process	GO:0019220	37	9.890	4.11E-15
	Regulation of phosphorus metabolic process	GO:0051174	37	9.890	4.11E-15
	Protein amino acid phosphorylation	GO:0006468	42	7.539	2.09E-14
	Regulation of kinase activity	GO:0043549	32	12.455	2.41E-14
	Regulation of transferase activity	GO:0051338	32	11.872	1.06E-13
	Response to organic substance	GO:0010033	48	5.738	2.16E-13
	Regulation of protein kinase activity	GO:0045859	30	12.280	1.81E-12
	Phosphorylation	GO:0016310	42	6.516	5.62E-12
	MAPKKK cascade	GO:0000165	24	18.618	9.75E-12
	Positive regulation of kinase activity	GO:0033674	26	14.494	5.94E-11
	Positive regulation of transferase activity	GO:0051347	26	13.866	1.81E-10
	Response to endogenous stimulus	GO:0009719	37	7.163	2.64E-10
	Positive regulation of molecular function	GO:0044093	34	7.439	6.39E-09
	Positive regulation of protein kinase activity	GO:0045860	24	13.939	8.79E-09
	Positive regulation of catalytic activity	GO:0043085	32	7.995	1.47E-08
	Response to hormone stimulus	GO:0009725	33	7.178	8.01E-08
	Regulation of apoptosis	GO:0042981	32	5.243	2.47E-06
	Regulation of programmed cell death	GO:0043067	32	5.174	3.54E-06
	Regulation of cell death	GO:0010941	32	5.152	4.01E-06
	Activation of protein kinase A activity	GO:0034199	9	66.561	3.21E-05
	Enzyme linked receptor protein signaling pathway	GO:0007167	21	8.320	7.94E-05
	Regulation of MAP kinase activity	GO:0043405	15	14.469	2.47E-04
	Cellular response to hormone stimulus	GO:0032870	16	12.588	2.76E-04
	Negative regulation of catalytic activity	GO:0043086	19	9.007	3.78E-04
	cAMP biosynthetic process	GO:0006171	8	73.957	4.80E-04
	Activation of protein kinase activity	GO:0032147	14	15.687	6.06E-04
Cellular constituent	Cytosol	GO:0005829	46	4.618	4.08E-17
	Cell fraction	GO:0000267	32	3.714	9.48E-08
	Nucleoplasm	GO:0005654	25	4.037	5.43E-06
	Insoluble fraction	GO:0005626	25	3.716	2.73E-05
	Soluble fraction	GO:0005625	16	6.026	5.40E-05
	Organelle lumen	GO:0043233	31	2.849	1.12E-04
	Plasma membrane	GO:0005886	45	2.108	1.93E-04
	Membrane-enclosed lumen	GO:0031974	31	2.772	2.06E-04
	Nuclear lumen	GO:0031981	26	3.148	3.24E-04
	Intracellular organelle lumen	GO:0070013	28	2.668	0.002417965
Molecular function	Protein kinase activity	GO:0004672	38	7.617	4.54E-12
	Purine ribonucleotide binding	GO:0032555	53	3.841	1.29E-11
	Ribonucleotide binding	GO:0032553	53	3.839	1.33E-11
	Protein serine/threonine kinase activity	GO:0004674	30	8.615	2.66E-11
	Purine nucleotide binding	GO:0017076	53	3.660	1.11E-10
	ATP binding	GO:0005524	46	4.169	6.83E-10
	Adenyl ribonucleotide binding	GO:0032559	46	4.087	1.47E-09
	Adenyl nucleotide binding	GO:0030554	46	3.856	1.44E-08
	Purine nucleoside binding	GO:0001883	46	3.789	2.78E-08
	Nucleoside binding	GO:0001882	46	3.759	5.66E-08
	Nucleotide binding	GO:0000166	53	3.059	3.25E-07
	MAP kinase activity	GO:0004707	9	59.748	7.78E-06
	Adenylate cyclase activity	GO:0004016	8	82.079	1.34E-05
	Phosphoprotein phosphatase activity	GO:0004721	15	13.329	5.76E-05
	Kinase binding	GO:0019900	16	10.031	5.56E-04

**Table 3:** KEGG pathway (FDR < 0.001, Fold enrichment > 2.0)

**KEGG_ID**	**Term**	**Count**	**Total number of genes in the pathway**	**Fold enrichment**	**FDR**	**Genes**
rno04010	MAPK signaling pathway	95	266	17.985	3.26E-19	RGD1565395, HRAS, PDGFA, TGFB3, NFKB1, FGF12, NFKB2, DAXX, TGFB1, TGFB2, AKT1, CDC42, FOS, CASP3, MOS, RRAS, PRKACA, PRKACB, FAS, MAP2K7, AKT3, AKT2, MAP2K5, EGFR, PRKCA, RELA, PTPRR, TP53, FLNC, ECSIT, FLNB, PRKCB, MAPK1, MAP4K4, RASGRF1, JUN, MAPK3, PDGFRA, HSPB1, MAPK9, PDGFRB, MAPK8, MAP3K14, GADD45A, FGFR2, FGFR1, TRAF2, GRB2, MRAS, MAPKAPK5, MKNK2, DUSP10, ELK1, HSPA1A, HSPA1B, MAPKAPK2, HSPA1L, TNFRSF1A, HSPA2, ELK4, SOS1, SOS2, DUSP16, RAC1, PPP3CB, PPP3CC, PPP3CA, CHP, HSPA8, PTPN7, MAP2K1, MAP2K2, PTPN5, MAP2K3, NLK, MAP2K4, TAOK3, NR4A1, RAF1, MAPK10, DUSP5, NRAS, DUSP4, DUSP3, ATF4, DUSP2, DUSP1, MAPK14, NTRK2, IKBKG, MAPK8IP3, DUSP9, IKBKB, CRK, DUSP7, DUSP6
rno04912	GnRH signaling pathway	42	94	22.501	1.01E-13	ADCY3, ADCY4, ADCY1, HRAS, ADCY2, ADCY7, GRB2, ADCY5, GNA11, ADCY6, ELK1, GNRHR, MMP2, CDC42, PTK2B, SOS1, SOS2, PRKACA, PRKACB, MAP2K7, EGFR, PRKCA, GNRH1, MAP2K1, MAP2K2, MAP2K3, MAP2K4, RAF1, MAPK10, MMP14, PRKCD, PRKCB, MAPK1, NRAS, ATF4, GNAQ, ADCY9, JUN, MAPK14, MAPK3, MAPK9, MAPK8
rno04540	Gap junction	29	81	18.030	9.06E-11	ADCY3, ADCY4, ADCY1, HRAS, ADCY2, ADCY7, PDGFA, GRB2, ADCY5, GNA11, ADCY6, SOS1, SOS2, PRKACA, PRKACB, MAP2K5, PRKCA, EGFR, MAP2K1, MAP2K2, RAF1, PRKCB, NRAS, MAPK1, ADCY9, GNAQ, MAPK3, PDGFRA, PDGFRB
rno04722	Neurotrophin signaling pathway	31	126	12.390	2.14E-09	HRAS, GRB2, NFKB1, MAPKAPK2, AKT1, CDC42, SOS1, RAC1, SOS2, MAP2K7, AKT3, AKT2, MAP2K5, MAP2K1, MAP2K2, RELA, TP53, RAF1, MAPK10, PRKCD, NRAS, MAPK1, ATF4, JUN, MAPK14, MAPK3, NTRK2, MAPK9, MAPK8, IKBKB, CRK
rno05200	Pathways in cancer	43	317	6.831	4.57E-08	FGFR2, TRAF2, FGFR1, HRAS, PDGFA, GRB2, TGFB3, NFKB1, FGF12, NFKB2, MMP2, TGFB1, TGFB2, AKT1, CDC42, FOS, CASP3, SOS1, SOS2, RAC1, FAS, AKT3, AKT2, EGFR, PRKCA, MAP2K1, MAP2K2, RELA, TP53, RAF1, MAPK10, PRKCB, MAPK1, NRAS, JUN, IKBKG, MAPK3, PDGFRA, PDGFRB, MAPK9, MAPK8, IKBKB, CRK
rno04662	B cell receptor signaling pathway	25	75	16.786	9.81E-07	HRAS, GRB2, NFKB1, AKT1, FOS, SOS1, RAC1, SOS2, PPP3CB, PPP3CC, CHP, PPP3CA, AKT3, AKT2, MAP2K1, MAP2K2, RELA, RAF1, PRKCB, NRAS, MAPK1, JUN, MAPK3, IKBKG, IKBKB
rno04660	T cell receptor signaling pathway	28	109	12.936	2.43E-06	HRAS, GRB2, NFKB1, AKT1, CDC42, FOS, SOS1, SOS2, PPP3CB, PPP3CC, PPP3CA, CHP, MAP2K7, AKT3, AKT2, MAP2K1, MAP2K2, RELA, RAF1, NRAS, MAPK1, JUN, MAPK14, IKBKG, MAPK3, MAPK9, IKBKB, MAP3K14
rno04062	Chemokine signaling pathway	32	171	9.424	2.04E-05	ADCY3, ADCY4, ADCY1, HRAS, ADCY2, ADCY7, GRB2, ADCY5, ADCY6, NFKB1, AKT1, CDC42, PTK2B, SOS1, SOS2, RAC1, PRKACA, PRKACB, AKT3, AKT2, MAP2K1, RELA, RAF1, PRKCD, PRKCB, NRAS, MAPK1, ADCY9, IKBKG, MAPK3, IKBKB, CRK
rno05215	Prostate cancer	25	90	13.988	1.32E-04	FGFR2, FGFR1, HRAS, PDGFA, GRB2, NFKB1, AKT1, SOS1, SOS2, AKT3, AKT2, EGFR, MAP2K1, MAP2K2, RELA, TP53, RAF1, NRAS, MAPK1, ATF4, IKBKG, MAPK3, PDGFRA, PDGFRB, IKBKB

### Immunohistochemical staining

**Figure 4:**
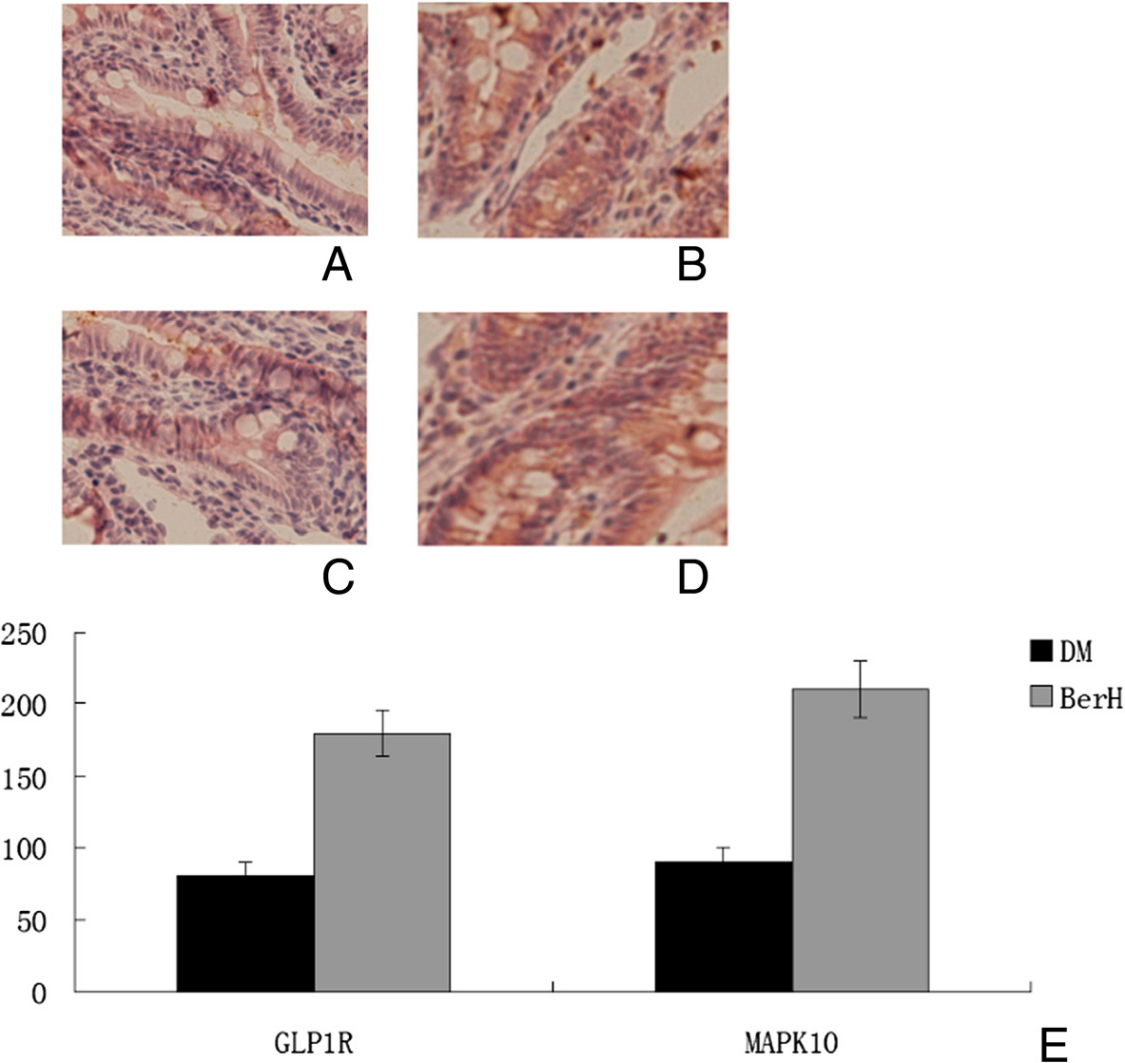
**The iluem immunohistochemistry for GLP1R and MAPK10 expression (original magnification × 200) and semiquantitative assessments. A**-**B** Immunostaining for GLP1R. **C**-**D** immunostaining for MAPK10. Iluem were harvested form DM **(A, C)** and berH group **(B, D)**. **(E)** Semiquatitatve scores of GLP1R and MAPK10. Data represent mean ± SD (n = 6). #*P* < 0.05 versus DM group.

To confirm that protein expression in the GLP1R and MAPK pathways was altered in the ileum of DM rats treated with berberine, immunohistochemistry analyses for GLP1R and MAPK10 were performed on ileum tissues. In the BerH group, there was a statistically significant increase in the immunoreactivities of GLP1R and MAPK10 (Figure 4).

### Q-RT-PCR experiment

We used Q-RT-PCR assays to verify some of the microarray results. Four genes [Glucagon-like peptide 1 receptor (Glp1r), gonadotropin-releasing hormone 1 (Gnrh1), gonadotropin releasing hormone receptor (Gnrhr) and mitogen activated protein kinase 10 (Mapk10)] were selected for verification, because of their central positions in the MAPK signaling pathway and GnRH signaling pathway. The expression ratios of these four genes, as determined through microarrays and Q-RT-PCR, are shown in Table 4. Glp1r and Mapk10 were up-regulated; while Gnrhr and Gnrh1 were down-regulated in both of the BerL group and BerH group. Strong agreement between the microarray and Q-RT-PCR results was observed for both genes, indicating the reliability of our microarray assays.

**Table 4:** Fold change in gene expression measured by gene array and Q-RT-PCR

**Gene symbol**	**BerL vs DM**			**BerH vs DM**		
	**Fold change (gene array)**	**Fold change (Q-RT-PCR)**	***P*_value (Q-RT-PCR)**	**Fold change (gene array)**	**Fold change (Q-RT-PCR)**	***P*_value (Q-RT-PCR)**
*Glp1r*	3.862	4.6 ± 0.3	0.027	4.231	4.3 ± 0.2	0.026
*Gnrh1*	−4.615	−3.7 ± 0.2	0.038	−3.758	−3.5 ± 0.3	0.025
*Gnrhr*	−3.715	−3.1 ± 0.4	0.014	−3.271	−2.9 ± 0.4	0.039
*Mapk10*	6.362	5.9 ± 0.3	0.031	5.837	6.2 ± 0.5	0.015

*Glp1r*: glucagon-like peptide 1 receptor; *Gnrh1*: gonadotropin-releasing hormone 1; *Gnrhr*: gonadotropin releasing hormone receptor; *Mapk10*: mitogen activated protein kinase 10.

Each Q-RT-PCR assay was repeated using three biological replicates and each analysis consisted of three technical replicates.

## Discussion

In this study, we found that the administration of berberine to diabetic rats significantly reduced fasting blood glucose, moderated glucose tolerance and reduced serum insulin. These results suggest that berberine can moderate glucose metabolism and ameliorate oral glucose tolerance and insulin sensitivity. These results are similar to the results of other studies [12-16].

Moreover, we found that berberine increased plasma GLP-1 after an oral glucose load. GLP-1 is a gut derived hormone secreted from intestinal L cells in response to glucose [17]. GLP-1 exerts important effects on the regulation of glucose metabolism, stimulating glucose-dependent insulin secretion and promoting β cell proliferation [18]. At the same time, GLP-1 inhibits glucagon release, gastric emptying and food intake [19]. Berberine may increase GLP-1 (7-36) amide secretion in STZ-induced diabetic rats [20], even in normal SD rats [21]. Recently, Shan et al. found that berberine could increase GLP-2 in type 2 diabetic rats [22].

We found that berberine could increase the expression of *Glp1r* in gene array experiments and Q-PCR experiments. Camilleri et al. found that GLP-1 can suppress gastrointestinal movement [23]. Furthermore, Feng et al. found that berberine could inhibit myoelectrical activity and gastrointestinal transit in rodents [21]. Therefore, berberine can increase plasma GLP-1 levels and Glp-1r expression to suppress gastrointestinal movement, moderate insulin secretion, and therefore moderate glucose metabolism.

In KEGG analysis, we found that the significant pathway in the berberine-treated group was the MAPK signaling pathway. Q-RT-PCR verified this result. TNF-α is interconnected with MAPK pathways. Waetzig et al. found that SB203580, a p38 inhibitor, significantly reduced mucosa secretion of TNF-α [24]. Hollenbach et al. found that SB203580 can reduce the mRNA levels of proinflammatory cytokines (i.e., TNF-α, IL-2 and IL-18) in the gut of BALB/c mice [25].

The GnRH signaling pathway was the second pathway in KEGG analysis. Q-PCR experiments also showed Gnrh1 and Gnrhr was reduced in the BerH group. Gonadotropin-releasing hormone (GnRH) and its receptor were expressed in the rat gastrointestinal system, pancreas and submaxillary glands, and its receptor had the same mRNA sequence with that of the hypothalamus, which demonstrated that GnRH was a brain-gut peptide [26,27]. Intestinal GnRH had a regulatory role on the endocrine and exocrine functions of the digestive system. GnRH and its receptor were also expressed in glucagon-immunoreactive cells of the rat ileum. GnRH analogs had a regulatory role on intestinal glucagon-like immunoreactivity [28]. A focused literature review showed GnRH agonists could increase the weight and fat mass and insulin resistance syndrome in men. These results indicate that GnRH may increase the risk of diabetes mellitus [29]. In premenopausal women with symptomatic uterine leiomyomas, GnRH agonists also increased insulin levels and HOMA scores [30]. Moreover, Antide, a GnRH receptor antagonist, administration prevented an increased incidence of diabetes in a castrated male nonobese mouse model of autoimmune diabetes (NOD) mice [31]. In addition, GnRH modulates the expression of insulin in the NOD mouse independently of gonadal steroids. These results demonstrated that GnRH could inhibit insulin secretion by islet cells *in vitro* experiments [32]. Thus, berberine may moderate blood glucose and insulin secretion through decreasing activity of GnRH.

## Conclusion

In conclusion, our studies provide evidence that berberine reduces blood glucose and insulin in diabetic rats. The mechanism may be within the MAPK pathway and GnRH-GLP-1 pathway in the ileum. These results provide molecular information for further investigation of the mechanisms by which berberine moderates glucose metabolism. Furthermore, these results could be important in devising mechanism-based and targeted therapeutic strategies for diabetes. More experiments should been performed in the future to confirm these results (such as studying gastrointestinal movement and use of Western blot analysis).

## Competing interests

The authors declare that there is no competng interest that could be perceived as prejudicing the impartiality of the research reported.

## Authors’ contributions

QZ, ZXW, MY, HBZ and FP conceived and designed the experiments and performed the experiments. ML, WHL and JZ analyzed the data. QZ wrote the manuscript. All authors read and approved the final manuscript.

## Pre-publication history

The pre-publication history for this paper can be accessed here:

http://www.biomedcentral.com/1472-6882/14/188/prepub
